# Analysis of aluminum toxicity in *Hordeum vulgare* roots with an emphasis on DNA integrity and cell cycle

**DOI:** 10.1371/journal.pone.0193156

**Published:** 2018-02-21

**Authors:** Joanna Jaskowiak, Oliver Tkaczyk, Michal Slota, Jolanta Kwasniewska, Iwona Szarejko

**Affiliations:** 1 Department of Plant Anatomy and Cytology, University of Silesia in Katowice, Katowice, Poland; 2 Department of Genetics, University of Silesia in Katowice, Katowice, Poland; Western Australia Department of Agriculture and Food, AUSTRALIA

## Abstract

Barley is one of the cereals that are most sensitive to aluminum (Al). Al in acid soils limits barley growth and development and, as a result, its productivity. The inhibition of root growth is a widely accepted indicator of Al stress. Al toxicity is affected by many factors including the culture medium, pH, Al concentration and the duration of the treatment. However, Al can act differently in different species and still Al toxicity in barley deserves study. Since the mechanism of Al toxicity is discussed we cytogenetically describe the effects of different doses of bioavailable Al on the barley nuclear genome—mitotic activity, cell cycle profile and DNA integrity. At the same time, we tested an established deep-water culture (DWC) hydroponics system and analyzed the effects of Al on the root system parameters using WinRHIZO software. We demonstrated the cytotoxic and genotoxic effect of Al in barley root cells. We showed that Al treatment significantly reduced the mitotic activity of the root tip cells and it also induced micronuclei and damaged nuclei. The DNA-damaging effect of Al was observed using the TUNEL test. We define the inhibitory influence of Al on DNA replication in barley. Analysis with the labelling and detection of 5-ethynyl-2‘-deoxyuridin (EdU) showed that the treatment with Al significantly decreased the frequency of S phase cells. We also demonstrated that Al exposure led to changes in the cell cycle profile of barley root tips. The delay of cell divisions observed as increased frequency of cells in G2/M phase after Al treatment was reported using flow cytometry.

## Introduction

Aluminum toxicity is considered to be the primary abiotic factor that limits crop production in regions with acid soils [1]. Aluminum is the most abundant metal and the third most abundant element in the earth’s crust and makes up 8% of its mass. In neutral pH, aluminum is bound in various minerals and among them bauxite is the most frequently occurring [2,3]. In soils with a pH level below 5.0, aluminum solubilizes and becomes available for plants as phytotoxic Al^3+^ ions [4]. Acid soils occupy more than 50% of the world’s arable land; they are predominant in the tropical and subtropical regions of South America, Central Africa and Southwest Asia, but they are also frequent in the temperate zones of eastern North America and Europe [5]. Additionally, the use of ammonia- and amide-containing fertilizers and industrial pollution promote soil acidification worldwide [6,7].

Trivalent aluminum ions (Al^3+^) inhibit cell proliferation and elongation by damaging root meristems. It has been shown that exposure to aluminum affects both the distal transition zone in a root [8] and the extensibility of the cell walls in the elongation zone [9]. At the cellular level, Al stress induces the depolarization of the plasma membrane, triggers an increase in cell wall rigidity and causes the disruption of the cytoskeleton [10], which adversely affects the uptake and transport of water and essential nutrients. Long-term exposure to Al may result in a deficiency of P, Ca, Mg, N and Fe and, as a result, cause an inhibition of plant growth and a decreased yield [5].

Although inhibition of root growth is one of the earliest and most dramatic symptoms exhibited by plants that are suffering from Al stress, the molecular mechanisms that underlies this phenomenon are still not fully understood. Studies in Arabidopsis have indicated that DNA is a primary target of Al and that a substantial increase in Al tolerance can be achieved by modifying the pathway that is responsible for monitoring DNA integrity [11,12]. The cytotoxic and genotoxic effects of Al have been observed in various plant species. Some of basic cytological symptoms of Al treatment, including mitotic activity and nuclear abnormalities, have also been studied in barley [13]. However, to the best of our knowledge, a detailed analysis of genotoxicity and cytotoxicity, especially using modern approaches, has not been performed in barley.

Among cereals, barley (*Hordeum vulgare* L.) is considered to be one of the most sensitive to Al toxicity [14–16]. Aluminum toxicity is the major factor that limits the production of barley on acid soils. There are several reports that describe the physiological effects of Al toxicity and genetic mechanisms that underlie the Al response [17, 18, 19]. The Al tolerance screening assays that were used in these studies differ in many respects, such as the methods of Al application, the Al concentration and duration of the treatment, the plant phenotypic trait that were analyzed and other details. The main genetic mechanism of resistance to Al^3+^ ions that have been described in barley is related to the excretion of the organic acids that enhance Al exclusion and prevent its uptake [20–22]. There is a lack of data on other molecular mechanisms that may lead to Al tolerance in barley that is similar to those reported in Arabidopsis [23]. In order to elucidate such mechanisms, it is necessary to first evaluate the cytotoxic and genotoxic effects of Al treatment in barley roots.

In this paper, we describe the effect of different doses of bioavailable Al on the root system parameters as well as on mitotic activity, the cell cycle profile and DNA integrity in barley. Using an established deep-water culture (DWC) hydroponics system, we showed that Al treatment resulted in a significant decrease in the mitotic activity of the root tip cells and an increased frequency of cells with micronuclei and damaged nuclei. The DNA-damaging effect of Al was also shown in a TUNEL test. Additionally, we demonstrated that after Al exposure, barley root tip cells changed their cell cycle profile and that they were predominantly in the G2/M phase.

## Materials and methods

### Plant material

The plant material used in the study was the spring barley (*Hordeum vulgare* L.) cultivar ‘Sebastian’ (parental line of the TILLING population that was developed at the Department of Genetics, University of Silesia). The preparation of plant material for Al treatment consisted of the sterilization of the barley seeds in a 5% solution of sodium hypochlorite (Sigma, Cat. no 71696) for 15 min, followed by rinsing them in sterile water (3 x 5 min). The seeds were placed in sterile square plastic 120 × 120 mm Petri plates (Gosselin, Cat. no BP124-05) covered with filter paper. The seeds were kept at 4°C for 24 h, transferred to an incubator for germination at 24°C for the next 24 h. The pregerminated seeds were used for the hydroponic culture.

### Al treatment

**Fig 1:**
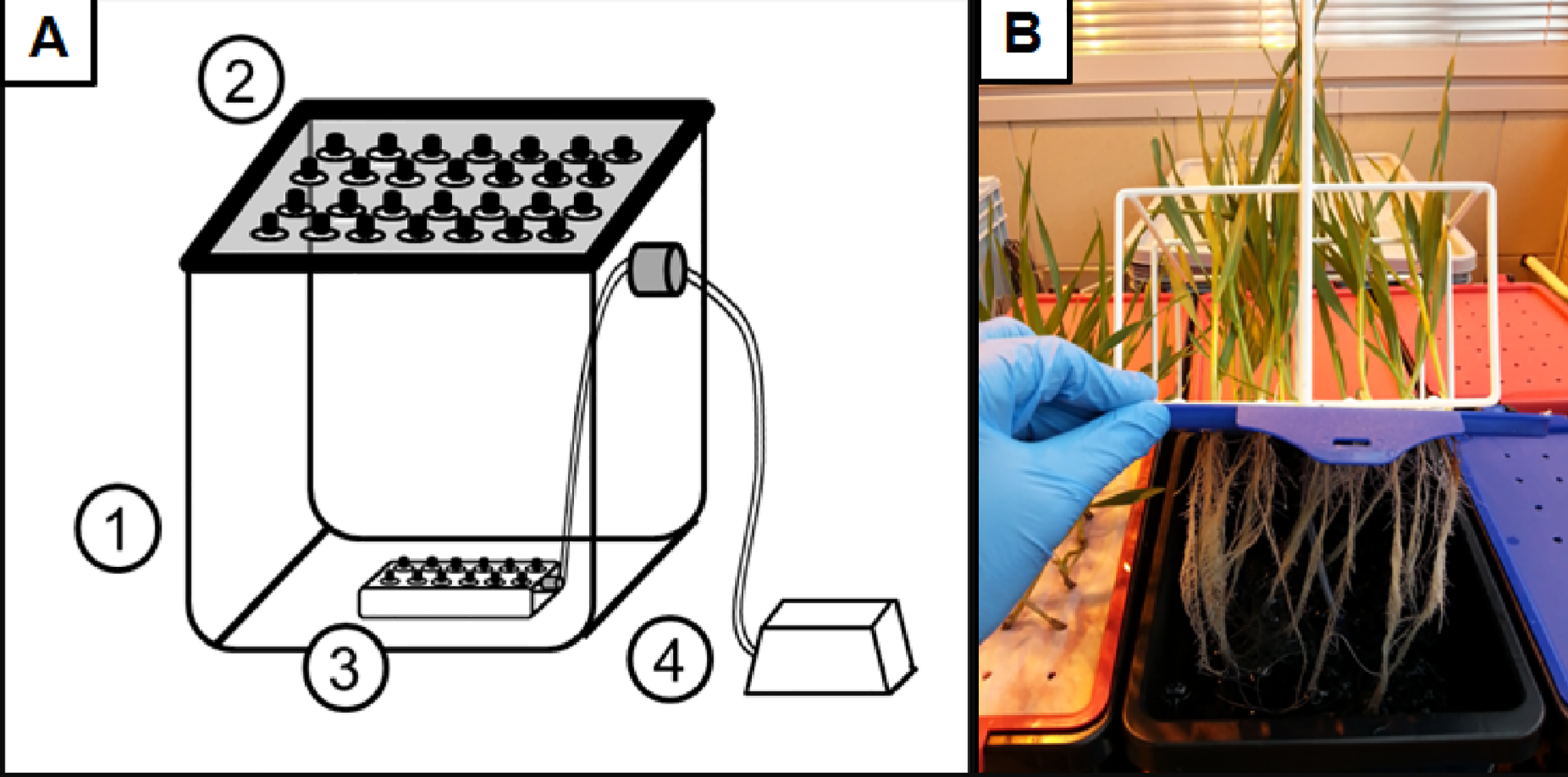
Schematic overview of the experimental setup for DWC hydroponics (A), and the photograph of the 7-day-old seedlings (B) grown in control conditions using that system (Hoagland’s medium, pH = 6). A plastic opaque container with capacity of 10 L (1), a plastic opaque lid with 20 openings (2), air distributor with 12 outlets (3) and an air pipe with a non-return valve attached to the air pump (4).

The setup for studying the effect of Al-induced stress on the root system parameters of different barley cultivars was based on deep-water culture (DWC) hydroponics (Fig 1).

The DWC hydroponics setup consisted of opaque plastic containers with a capacity of 10 L with opaque plastic lids with 20 openings, air distributors with 12 outlets and air pipes with a non-return valve attached to the air pump (with an air flow of 640 L/min). In the Al-treatment experiments, a classical full Hoagland’s nutrient solution was used as the hydroponic medium [24]. The bioavailable Al^3+^ concentrations in the nutrient solution were calculated using GEOCHEM-EZ software [25]. In the presented study, the indicated AlCl_3_ concentration always refers to its bioavailable fraction, unless stated otherwise. Al was applied to the hydroponic medium as the appropriate amount of 1 M AlCl_3_ solution. The pH of the hydroponic medium was adjusted to 4.0 for the control assay and Al treatments. In order to determine whether the changes in pH affected the cell cycle and S-phase in the control, the pH of the hydroponic medium was adjusted to 6.0. The pH of the hydroponic cultures was determined and adjusted every day using a 1 M NaOH or 1 M HCl solution. The Al concentrations that were tested were 5, 10, 20, 40, 60 μM AlCl_3_ for the optimizing experiments and a narrower spectrum of doses– 20, 30, 40 μM AlCl_3_ for the final Al treatments (Table 1).

**Table 1:** AlCl_3_ concentrations selected for the treatment of barley seedlings grown in DWC hydroponics. The calculations of available Al^3+^ concentrations in the full Hoagland’s nutrient solution, pH = 4 were calculated using GEOCHEM-EZ software.

Concentration	Tested aluminium doses					
Nominal {Al^3+^}* [mM]	0.125	0.25	0.5	0.75	1	1.5
Nominal {Al^3+^}* [ppm]	3.375	6.75	13.5	20.25	27	40.5
**Available Al**^**3+**^ **[μM]**	**5**	**10**	**20**	**30**	**40**	**60**
**Available Al**^**3+**^ **[ppm]**	**0.135**	**0.27**	**0.54**	**0.81**	**1.08**	**1.62**

{Al3+}*—a nominal dose of applied AlCl_3_ concentration in a full Hoagland’s nutrient solution

The initial selection of the Al concentrations was based on the available literature data on the Al treatment of barley [26–31]. The maximal concentration of AlCl_3_, which was applied, was 60 μM and the minimal concentration was 5 μM. The latter was applied with the intention of determining the concentration that would be appropriate to assess Al hypersensitivity.

The germinated barley seeds were implanted into the openings on lids that were covered with moistened filter paper. Three biological replicates in individual containers were set up, each containing 20 seedlings. In the experiment to measure the growth dynamics, the root systems of the barley seedlings were scanned after 48 h, 96 h and 144 h of aluminum exposure. The DWC hydroponic systems were kept in a growth chamber under controlled conditions—temperature 22/20°C during the day/night, photoperiod 16/8 h and light intensity of 320 μmol m^−2^ s^−1^. The growth of the control and Al-treated plants was carried out for 1 and 7 days.

### Analysis of root growth

After treatment, the plant roots were immediately scanned in an aqueous solution or were preserved in a 50% ethyl alcohol solution in 50 ml Falcon flasks. The root system scanning was performed using a specialized root scanner (STD4800 Scanner) and WinRHIZO Pro software (Regent Instruments). The roots were cut using sharp scissors in order to separate them and then placed on a waterproof tray in water (Regent Instruments). The roots were spread out on the trays in order to avoid any overlapping lateral roots and to ensure a random distribution. The parameters that were generated using the WinRHIZO system included the total length of the root system (cm), the root system surface (cm^2^), the root system volume (cm^3^) and the average root diameter (mm). Statistical analyses of the parameters that characterize the root systems were performed using an ANOVA analysis (P < 0.05) followed by a Tukey’s honest significant difference test (Tukey HSD test).

### Analysis of mitotic activity, the frequency of cells with micronuclei and damaged nuclei

Some of the material that had been treated with 20, 30 and 40 μM AlCl^+3^ for 1 and 7 days as well as the control material were used for the cytogenetic studies. The mitotic activity of the meristematic barley root cells and the frequency of cells with micronuclei and damaged nuclei were analyzed. The roots were fixed in methanol: acetic acid (3:1 v/v) for 4 h at room temperature (RT). Cytogenetic slides were prepared using the Feulgen’s squash technique. In each experimental combination, the cytogenetic parameters listed above were counted for 2000 cells.

### TUNEL test

The TUNEL (terminal deoxynucleotidyl transferase-mediated dUTP nick-end labeling) reaction was used to analyze any Al-induced DNA fragmentation. Control roots and roots that had been treated with 20, 30 and 40 μM AlCl^+3^ for 1 and 7 days were fixed in freshly prepared 4% paraformaldehyde (Fluka) in PBS (phosphate-buffered saline) for 1 h at RT and then washed 3 x 5 min in PBS. The nuclei preparations were prepared by squashing the meristematic tissue in the PBS buffer. After freezing at -70 °C, the slides were stored at 4°C for several days. Prior to the TUNEL reaction, the slides were air dried, permeabilized by incubating in 0.1% Triton X-100 (Sigma) in 0.1% sodium citrate at 4°C for 2 min and were then rinsed in PBS. For the positive control, a slide was treated with a DNase solution (1U) for 30 min at 37°C in a humid chamber. DNA fragment labeling was carried out using a TUNEL reaction mixture (*in situ* Cell Death Detection Kit, Fluorescein, Roche) containing an enzyme solution (terminal transferase) and a label solution (FITC-labeled nucleotides). Fifty μl of the TUNEL reaction mixture (enzyme solution: label solution, 1:9 v/v) was applied to the preparations and incubated in a humid chamber for 1 h at 37°C in the dark. As a negative control of the TUNEL reaction, a reaction mixture without any enzyme was used. The preparations were rinsed 3 x in PBS and stained with DAPI (2 μg/ml), air dried and mounted in a Vectashield medium (Vector Laboratories). The frequency of TUNEL-positive nuclei was analyzed. The frequency of FITC-labeled nuclei in the TUNEL test was established based on an analysis of 2000 cells on each of two slides (each prepared from one root meristem) for the one treatment experiment. For the combination, two Al treatment experiments were used. A total 8000 nuclei were analyzed for the combination. Two independent treatment experiments were carried out for all of the experiments as specified for each method. Preparations were examined using a Zeiss Axio Imager.Z.2 wide-field fluorescence microscope equipped with an AxioCam Mrm monochromatic camera.

### Cell cycle analysis using flow cytometry

For the cell cycle analysis with a flow cytometer, material that had been treated with the highest Al concentration– 40 μM AlCl^+3^ for 1 and 7 days was used. For each experimental combination, approximately 30–50 root meristems were analyzed. After mechanical root tip fragmentation, the suspension of nuclei was filtered through a 30-um nylon mesh to remove any large debris and then stained with a staining buffer (CyStain^®^ UV Precise P, 05–5002, Sysmex). Samples were incubated for 1–2 minutes and analyzed using a CyFlow Space Sysmex flow cytometer with a 365 nm UV LED diode as the light source. Two samples were analyzed for each experimental group and the flow rate was adjusted to 20–40 nuclei per second. The results are shown on histograms that were prepared using a linear scale. To determine the cell cycle phase, FloMax software with the Cell Cycle Analysis application was used.

### S-phase analysis with EdU

Detailed data on the frequency of the cells in S-phase were obtained by incorporating EdU (5-ethynyl-2`-deoxyuridine; Click-iT EdU Imaging Kits Alexa Fluor 647, Invitrogen). The control roots and roots that had been treated with 20, 30 and 40 μM AlCl^+3^ for 1 and 7 days were incubated in a 10 mM EdU solution for 1 h in the dark. After the incorporation of EdU, the seedlings were rinsed in distilled water 2 x for 5 min and fixed in 3.7% paraformaldehyde in PBS for 30 min. The fixed seedlings were washed 3 x for 5 min in PBS. To prepare the nuclei, the roots of the seedlings were washed with a 0.01 mM sodium citrate buffer (pH 4.8) for 30 min and digested with 2% cellulase (w/v, Onozuka, Serva) at 37°C for 1 hour. After digestion, the material was washed again in a sodium citrate buffer for 30 min. Squash nuclei preparations were prepared in a drop of PBS. After freezing and removing the coverslips, the slides were dried. Prior to EdU detection, the slides were permeabilized with 0.5% Triton X-100 for 20 min and then washed in PBS at RT. The slides were incubated for 30 min at RT in an EdU reaction cocktail (Click-iT EdU Imaging Kits Alexa Fluor 647, Invitrogen), which was prepared according to the manufacturer’s procedure. For one sample reaction, the following components were added: 43 μl of a 1 x Click-iT reaction buffer, 2 μl of CuSo_4_ (Component E, 100 mM), 0.12 μl Alexa Fluor 647 azide (Component B) and a 5 μl reaction buffer additive (Component F). After two 5 min washes, the slides were stained with 2 μg/ml DAPI (Sigma), washed in PBS and mounted in a Vectashield medium (Vector). The frequency of the EdU-labeled nuclei in the S-phase were counted. The frequency of nuclei in the S-phase was established based on analysis of 2000 cells on each of two slides (each prepared from one root meristem) for the one treatment experiment. For the combination, two Al treatment experiments were performed. In total 8000 nuclei were analyzed for the combination. Two independent treatment experiments were carried out for all of the experiments as specified for each method. Preparations were examined using a Zeiss Axio Imager.Z.2 wide-field fluorescence microscope equipped with an AxioCam Mrm monochromatic camera.

Statistical analyses to calculate the mitotic activity, the frequency of cells with micronuclei and damaged nuclei, the frequency of the S-phase cells and the frequency of DNA-damaged cells were performed using an ANOVA analysis followed by the Student’s t-test or a Tukey’s honest significant difference test (Tukey HSD test) with the p-values that are indicated in the figure legends.

## Results

### Growth root parameters

The optimizing Al treatments that were conducted consisted of:

1. a detailed analysis of the parameters of the root systems after 7-day treatment of barley seedlings with a range of aluminum concentrations (5, 10, 20, 30, 40, 60 μM),
2. an analysis of the effect of Al treatment on the dynamics of root growth using measurements of the root system parameters at selected time points (0, 2, 4, 6 days).

**Fig 2:**
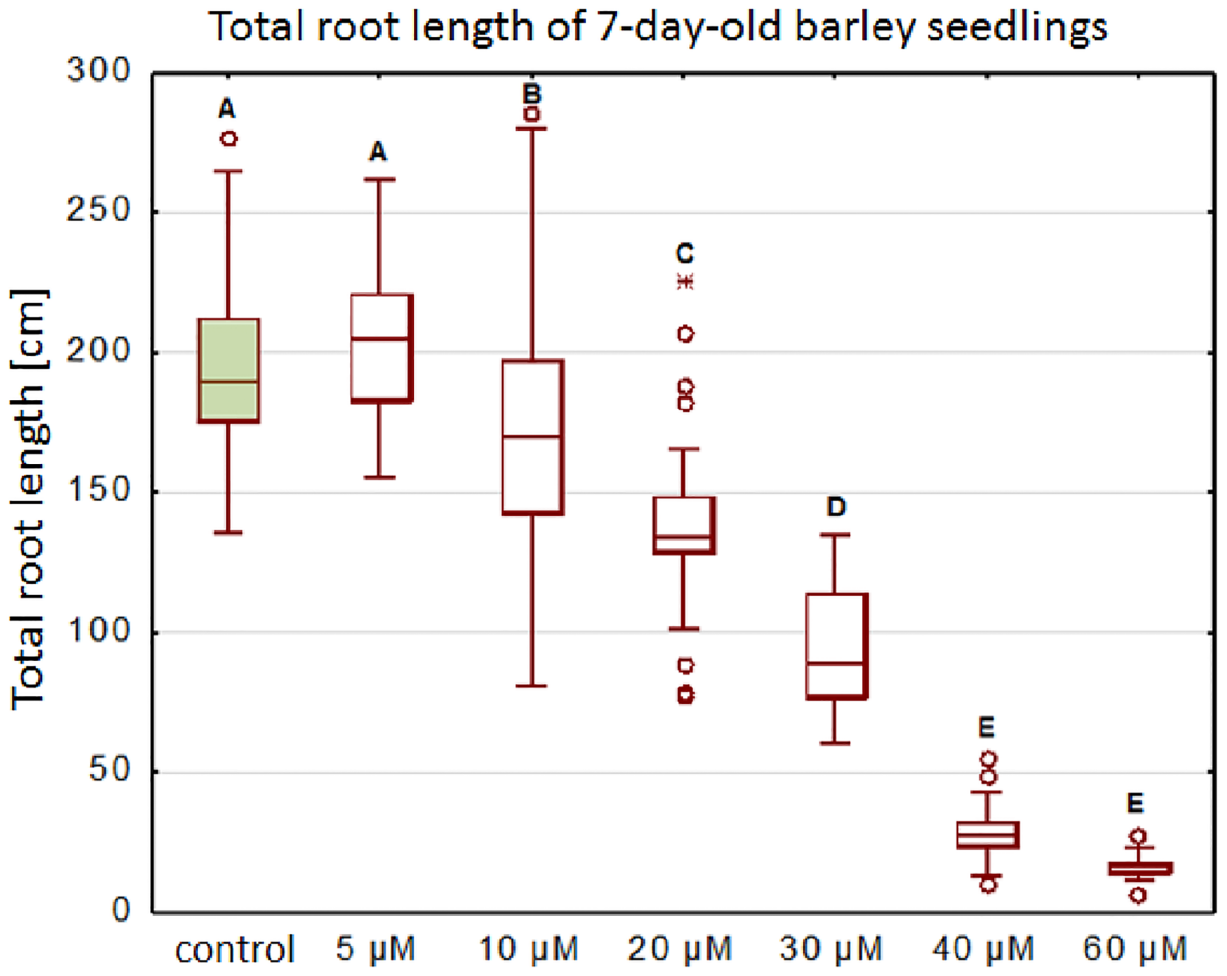
The total root length of barley seedlings cv. ‘Sebastian’ treated with different concentrations of Al for 7 days. Tested Al^3+^ doses included 5; 10; 20; 40; 60 μM AlCl_3_, in full Hoagland’s medium, pH = 4 (treatments), pH = 6 (control). The measurements were carried out using WinRHIZO system.

A significant decrease in the total root length of the seedlings was observed in all of the tested concentrations after seven days of Al treatment, except for the 5 μM AlCl_3_ (Fig 2). About a 50% decrease in the total root length was observed for the seedlings that had been exposed to the concentration of 30 μM AlCl_3_. It was shown that a further increase of the aluminum concentration drastically affected the plant roots and caused an almost complete inhibition of their growth, up to a 92% decrease in the total root length after exposure to 60 μM AlCl_3_.

**Fig 3:**
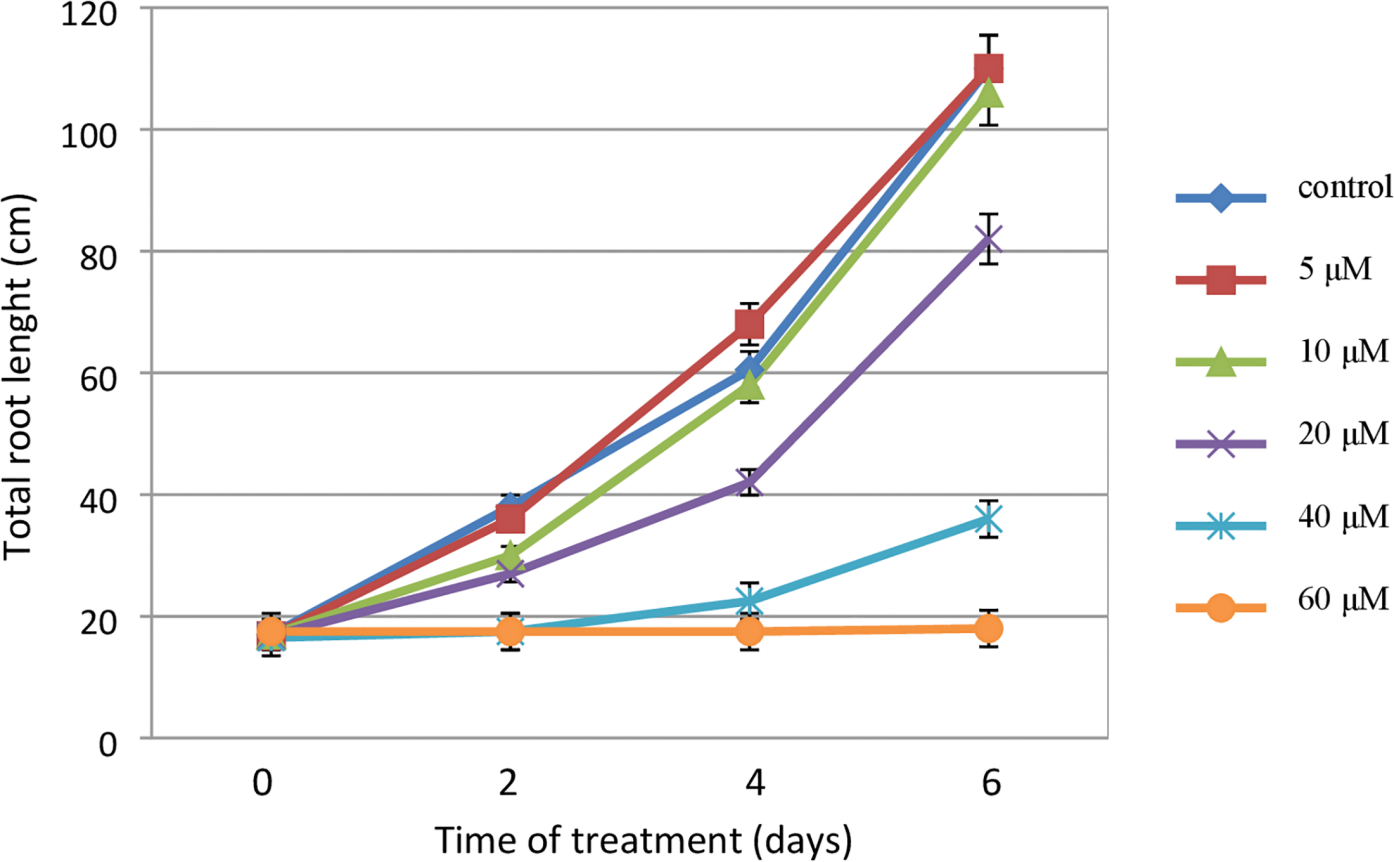
The kinetics of root growth of barley seedlings cv. ‘Sebastian’ treated with different concentrations of Al. The error bars represent the standard deviations of the mean. Tested Al doses consisted of: 5; 10; 20; 40; 60 μM AlCl_3_, in full Hoagland’s medium, pH = 4 (treatments), pH = 6 (control). The total root length was analysed at selected time points of continuous Al-treatment: 0 days; 2 days; 4 days; 6 days. The measurements were carried out using WinRHIZO system.

Further analyses were conducted in order to assess the effect of the aluminum treatment on root growth over time. An analysis of the dynamics of the root growth of barley seedlings indicated that the effect of Al treatment could already be observed after 2 days (48 h) of exposure. The toxic effect of exposure to Al was stronger after 4 and 6 days of treatment and also depended on the concentration that had been used. The dynamics of root growth was similar for the untreated plants and the plants that had been exposed to the two lowest doses of Al^3+^ (5 and 10 μM), while it differed significantly for the plants that had been treated with a concentration of 20 μM AlCl_3_ and those that had been exposed to the highest doses (Fig 3).

**Fig 4:**
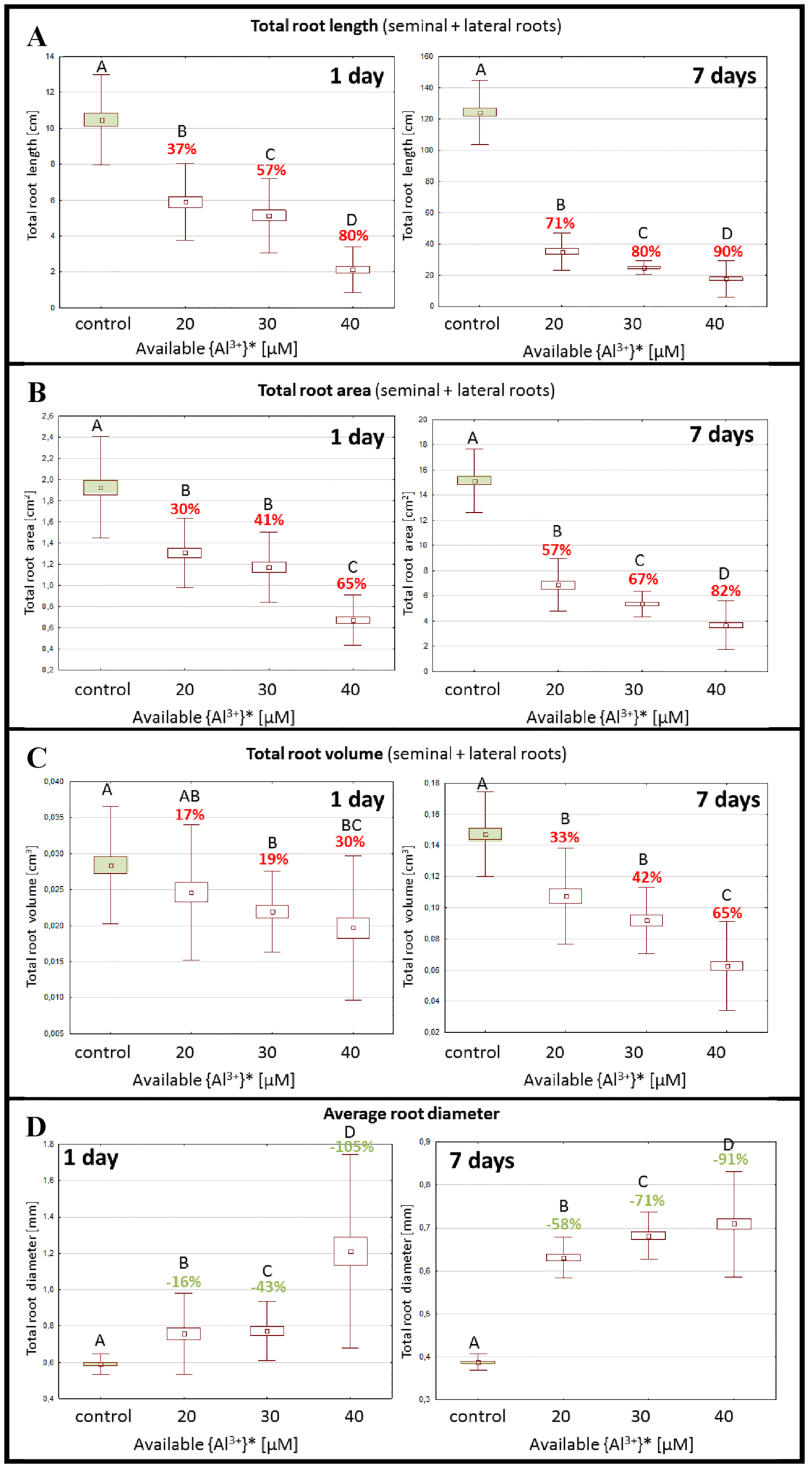
The effect of the Al-treatment on root system parameters evaluated after 1- day and 7 days of the exposition. Tested Al^3+^ doses consisted of: 10; 20; 30 μM AlCl_3_, in full Hoagland’s medium, pH = 4 (treatments), pH = 6 (control). The measured parameters include: total root length (A), total root area (B), total root volume (C), average root diameter (D). Different letters above the bars indicate statistically significant differences (ANOVA; p < 0.05) between combinations. The percentages above the bars indicate the reduction/ increase of the value in comparison to the control.

**Fig 5:**
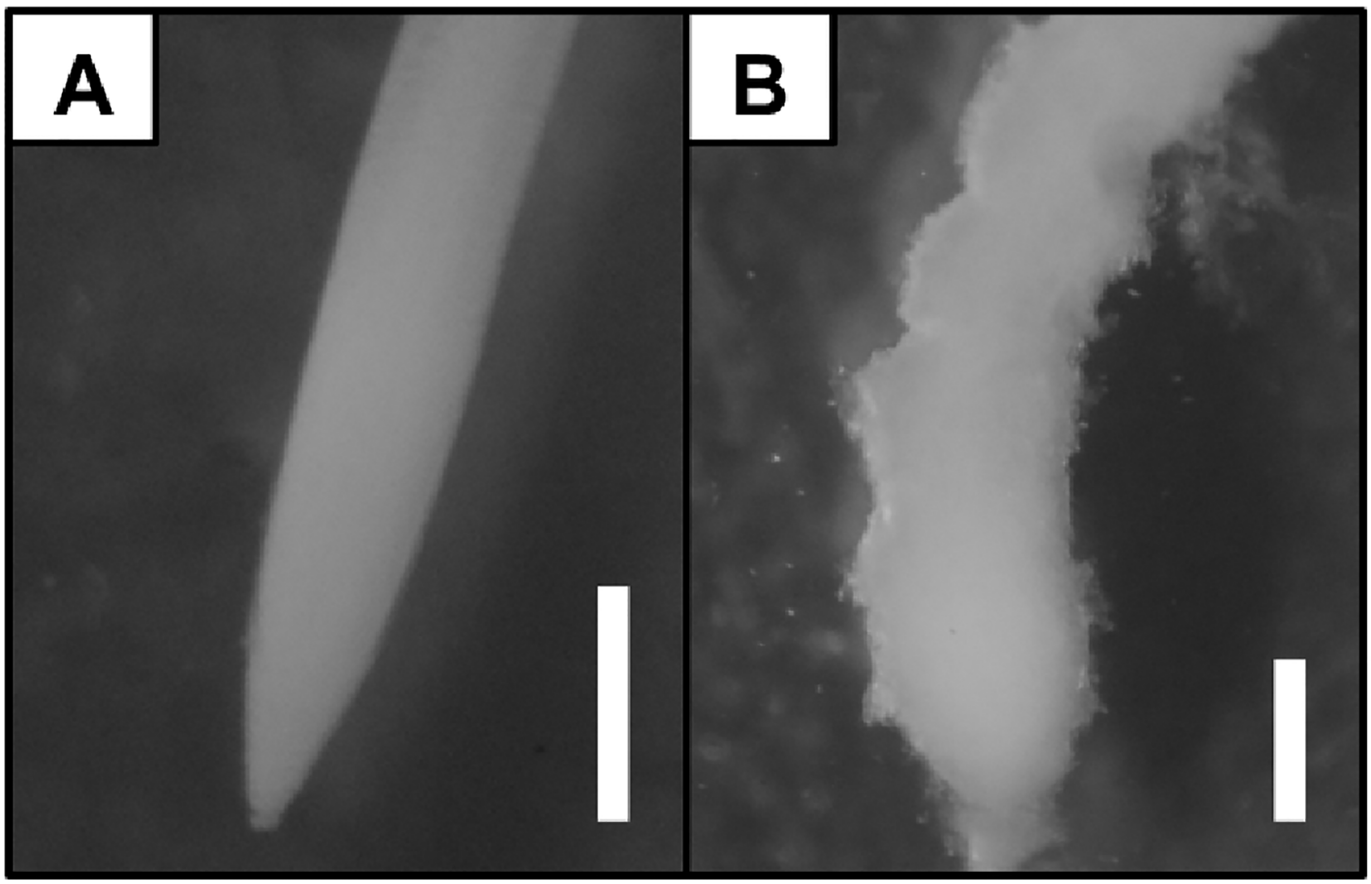
The root apices of barley seedlings (cv. ‘Sebastian’) grown in DWC hydroponics in full Hoagland’s control medium (A), and exposed to 40 μM AlCl_3_ (B). The photographs were taken using Delta Optical SZ-630T stereomicroscope, scale bar = 0.5 mm.

Based on the preliminary experiments, a comprehensive assessment of the effect of Al treatment on the parameters of the root system was conducted. For this purpose, three aluminum concentrations– 20, 30 and 40 μM were selected. The effect of the Al treatment was evaluated after 1 day and 7 days of exposure. The treatment of the barley seedlings with the lowest Al concentration (20 μM AlCl_3_) already caused a significant alteration in the parameters of the root system after one day of exposure and was much stronger after 7-days of exposure to Al (Fig 4). The most profound effect of Al was observed for the parameter for the total root length. The decrease of this parameter for the Al concentrations that were tested ranged from 37% to 80% after 1 day and 71% to 90% after 7 day treatment, respectively. A similar strong decrease was observed for the parameter for the total root area, while the total root volume was less affected and reached 30% after 1-day and 65% after 7-days of treatment with the highest Al concentration (40 μM AlCl_3_). This effect was associated with a significant increase in the average root diameter and was observed for all aluminum doses (Fig 4) That in turn may be related to the decrease of root growth and the ‘stubby and brittle’ phenotype of the roots that had been exposed to Al (Fig 5).

### Cytological effects of Al

**Fig 6:**
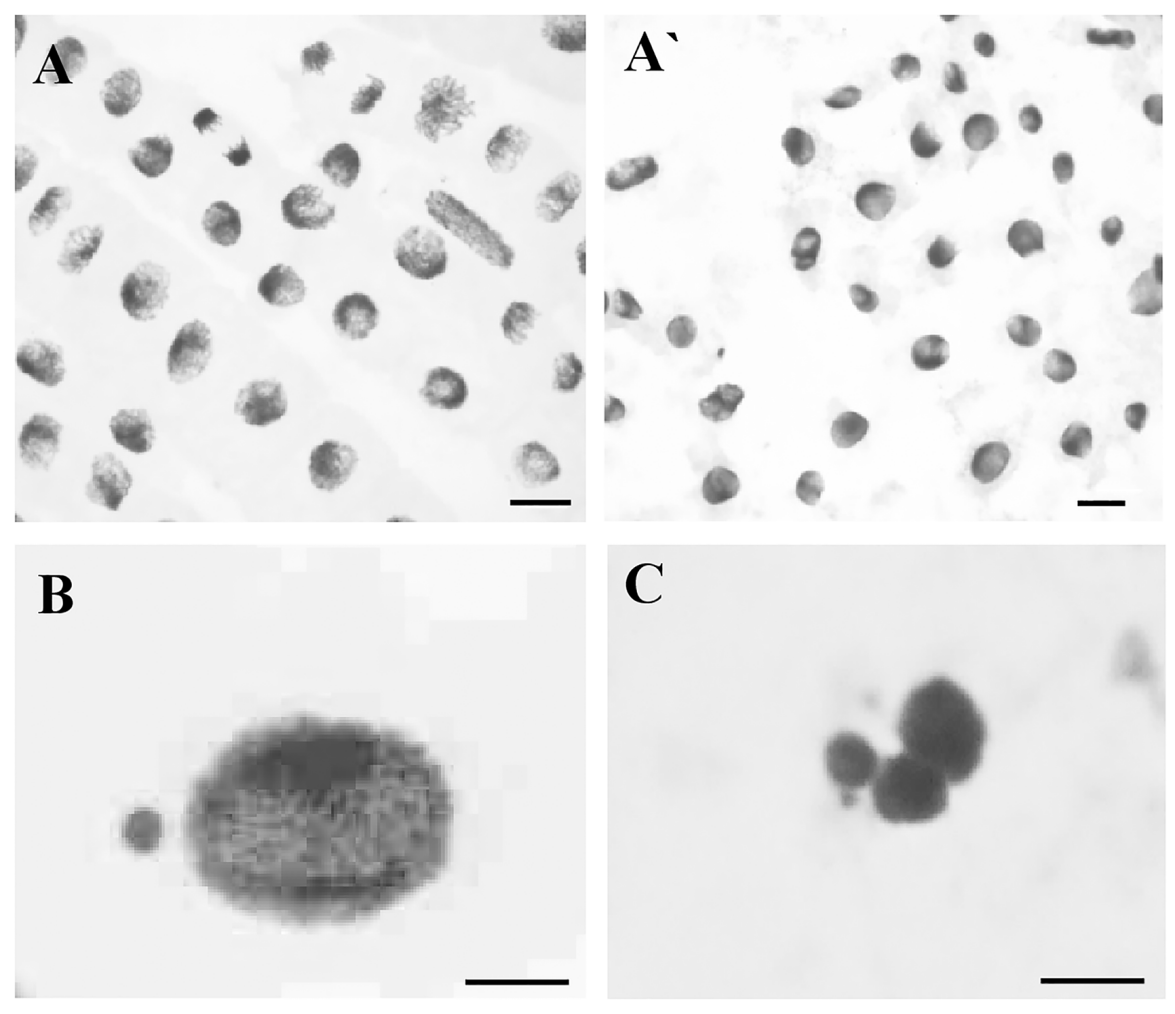
Cytological effects of Al in root cells of barley seedlings cv. ‘Sebastian’. (A, A`) mitotic activity of control cells (A) and cells after Al^3+^ treatment (A`), (B) interphase nucleus with micronucleus, (C) fragmented interphase nuclei. Bars represent 20 μm.

**Fig 7:**
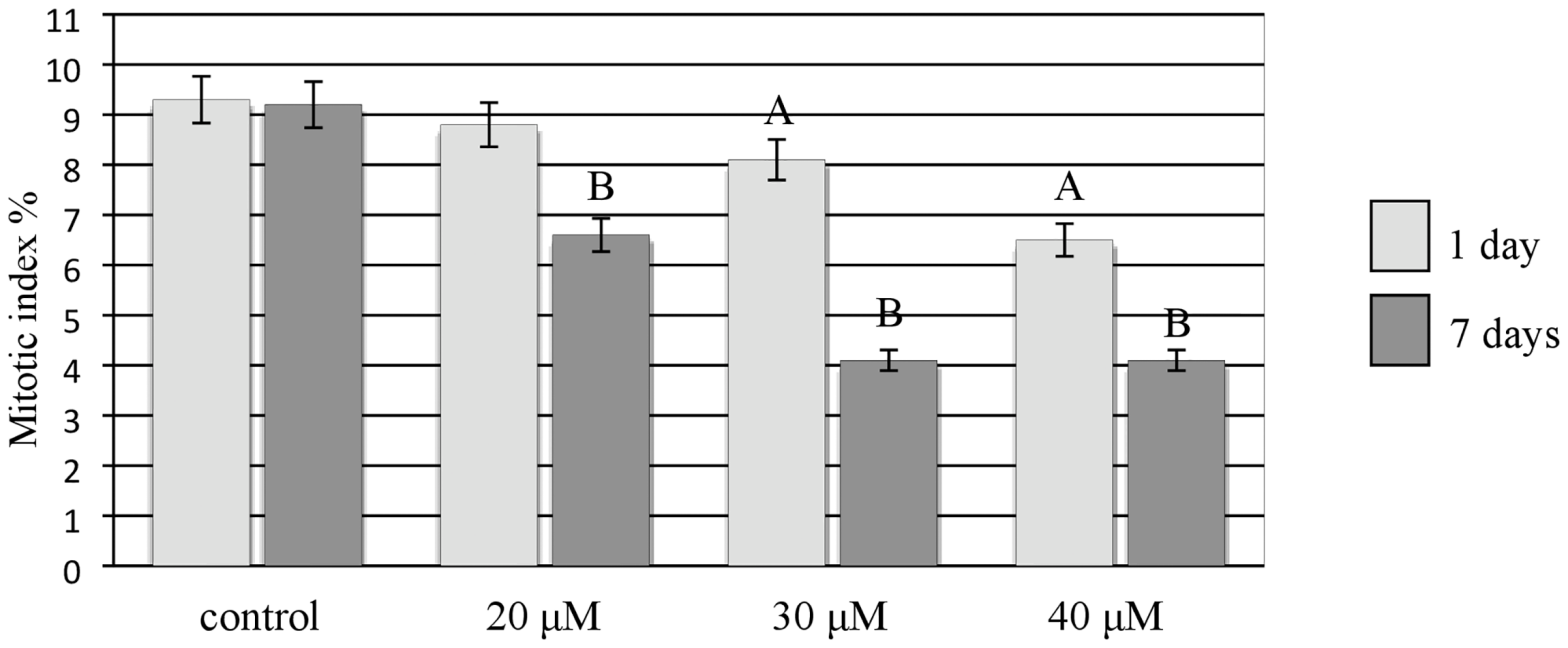
The mitotic activity of root cells of barley seedlings cv. ‘Sebastian’—(control and Al treated). The error bars represent the standard deviations of the mean. The significant differences (P < 0.05) between the treated combinations and the control within the same treatment day, are indicated by a capital letter A (1-day treatment) or B (7-day treatment).

The Al treatment of the barley cv. ‘Sebastian’ roots also caused cytological effects such as a decrease in mitotic activity, micronuclei and damaged nuclei and their disintegration (Fig 6A, 6A`, 6B and 6C). Although all of the concentrations of Al that were used for the treatments– 20, 30, 40 μM reduced mitotic activity, the effects strongly depended on the duration of the treatment (Fig 7). The lowest mitotic activity (4.1%) was observed for the highest Al concentration, whereas the frequency of dividing in the control was 9.3%. A significant decrease in mitotic activity was shown for 30 μM and 40 μM of Al for the 1-day treatment. All of the concentrations of Al significantly decreased the mitotic activity of root cells for the 7-day treatment. After 7-day treatment with 30 and 40 μM Al, the frequencies of dividing cells were the same.

**Fig 8:**
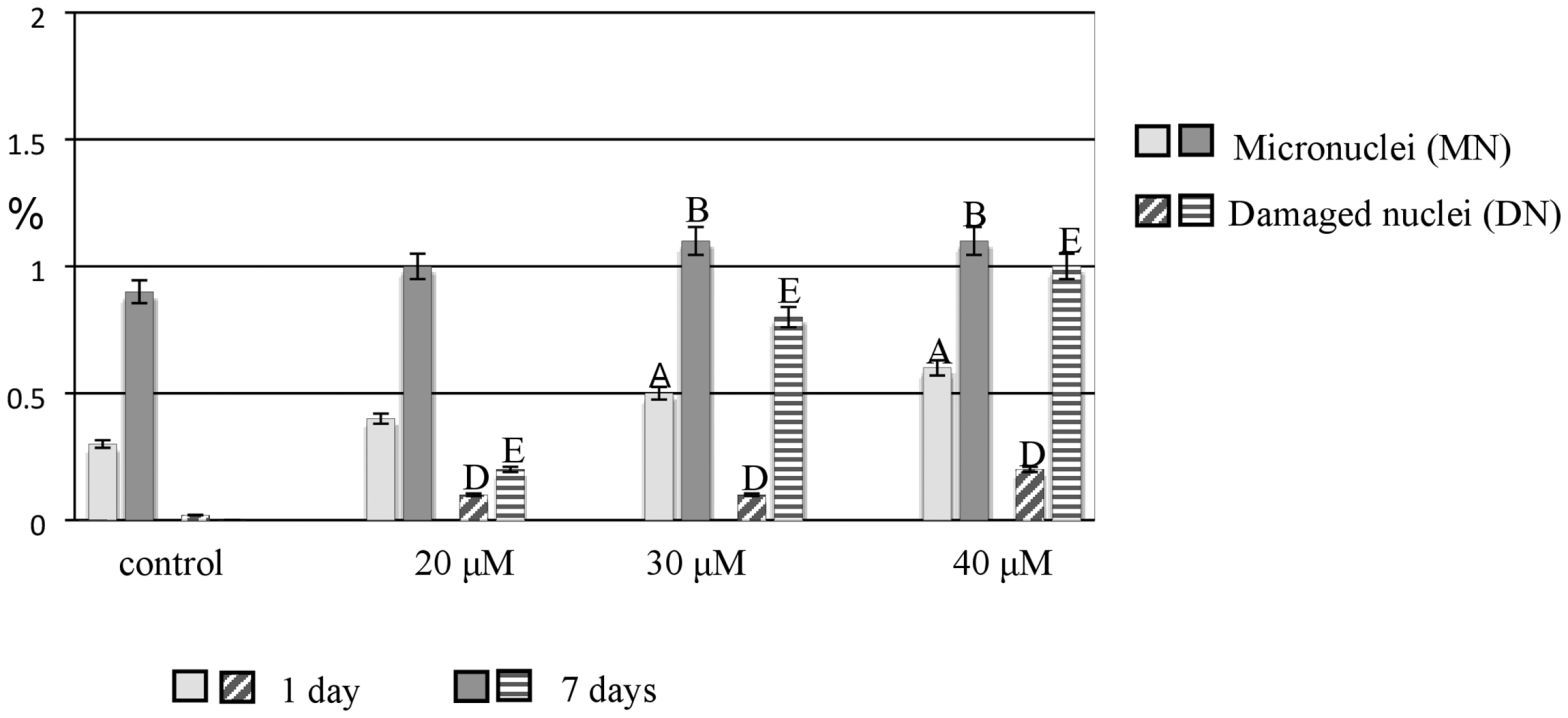
The frequency of root cells of barley cv. ‘Sebastian’ with micronuclei and damaged nuclei in control and after Al treatment. The error bars represent the standard deviations of the mean. The significant differences (P < 0.05) between the treated combinations and the control within the same treatment day are indicated by capital letters: A, B, D, E.

One of the cytogenetic effects of Al treatment in the barley meristematic root cells was the formation of micronuclei. Although micronuclei were also observed in the control, their frequency was much higher in the Al-treated roots. All of the applied concentrations of Al induced micronuclei. A significant increase in the frequency of micronuclei was observed for 30 μM and 40 μM Al after both the 1- and 7-day treatments. The other observed cytogenetic effects of Al treatment were disrupted nuclei. While no such effect was observed in the control, damaged nuclei with a frequency of 1% were already observed after the 1-day treatment with 20 μM and 7-day exposure to 40 μM Al (Fig 8).

### DNA damage

**Fig 9:**
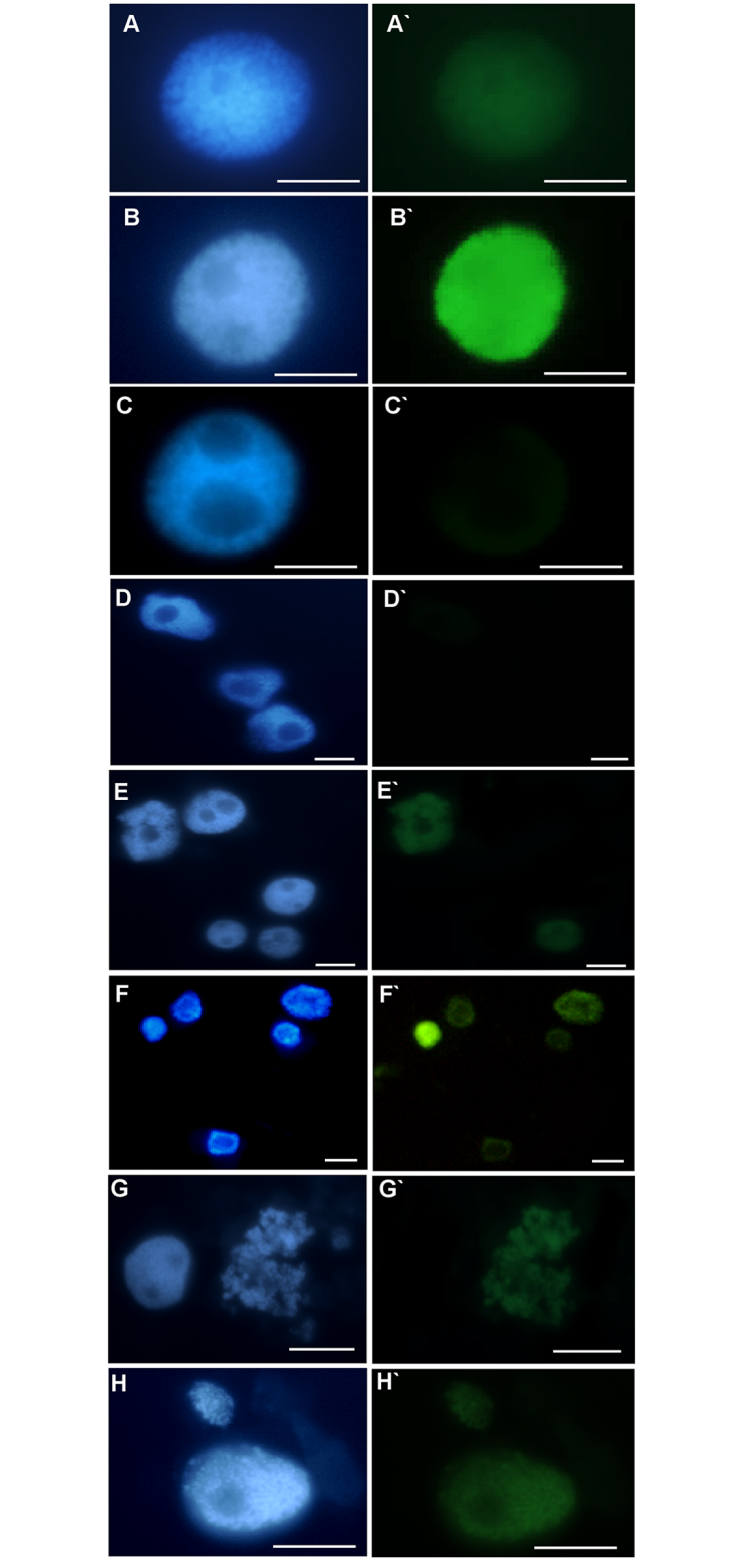
Results of TUNEL test in root cells of barley seedlings cv. ‘Sebastian’. DAPI stained nuclei (A-H), with or without green fluorescence as a result of TUNEL reaction (A`-H`). (A, A`) control nuclei showing weak green fluorescence, (B, B`) positive control (DNAse solution was used to induce DNA strand breaks), (C, C`) negative control (nucleotide solution, without terminal transferase was used), (D, D`) not-treated cells, (E, E`) after treatment with 30 μM Al (nuclei with and without green fluorescence are shown), (F, F`) after treatment with 40 μM Al (nuclei with and without green fluorescence are shown) (G, G`) fragmented nucleus after treatment with 30 μM Al with green fluorescence, (H, H`) nuclei with micronucleus, both show green fluorescence. Bars represent 20 μm.

**Fig 10:**
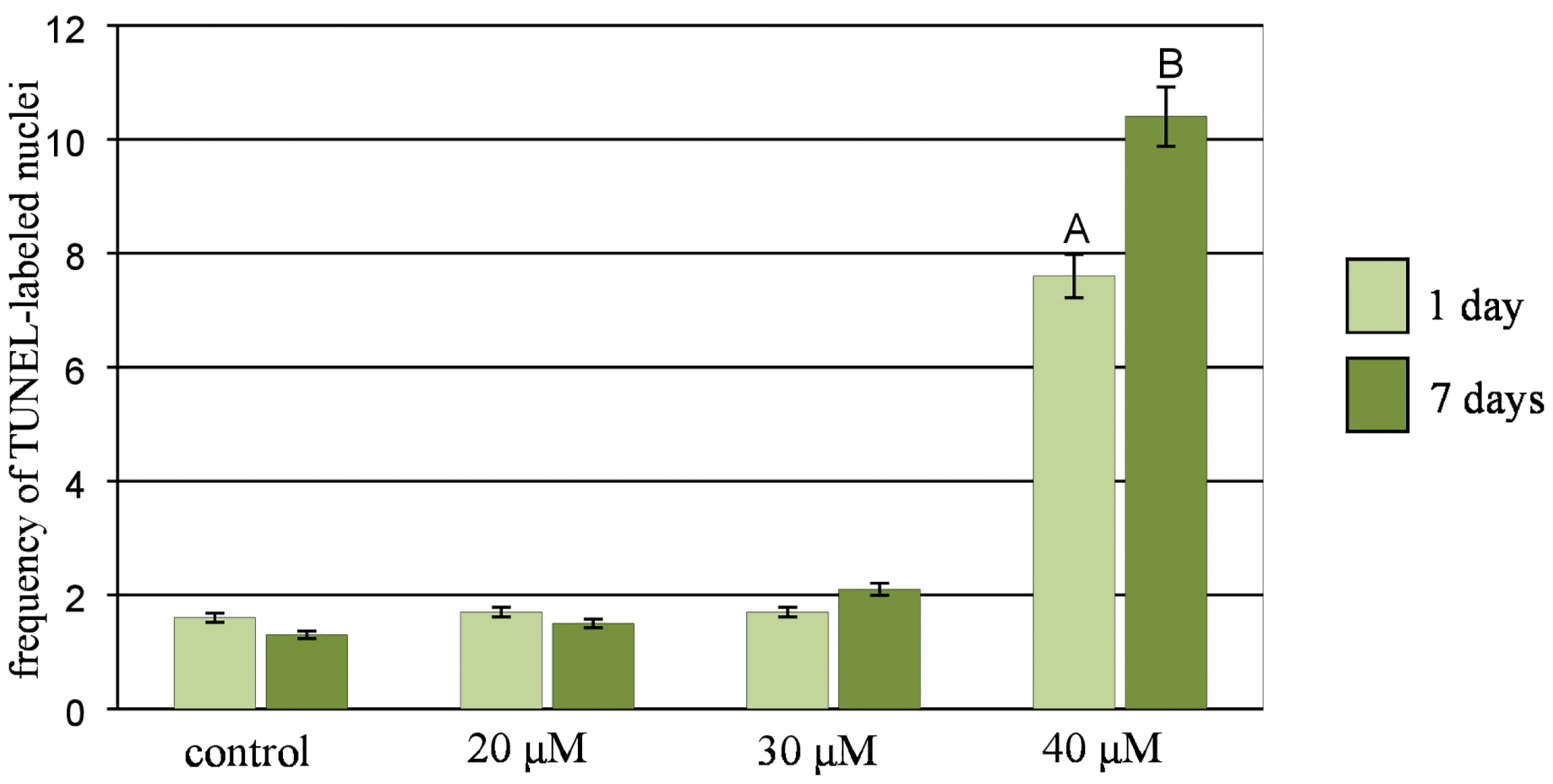
The frequency of labelled nuclei in root cells of barley cv. ‘Sebastian’ in TUNEL test in control and after Al treatment. The error bars represent the standard deviations of the mean. The significant differences (P < 0.05) between the treated combinations and the control within the same treatment day are indicated by a capital letter A (1-day treatment) or B (7-day treatment).

To assess the nuclei with DNA nicks and DNA fragments in the control and Al-treated barley roots, the TUNEL test was applied. To precisely determine the percentage of damaged nuclei, all of the cells were stained with DAPI (Fig 9A–9H). The same nuclei, which had a green fluorescence, that were observed in the FITC channel were characterized by DNA damage (Fig 9A`–9H`). In the control cells, TUNEL-specific nuclei with relatively weak fluorescence (Fig 9A and 9A`) were observed with a frequency of about 1.5% (Fig 10). The material that had been treated with DNase (positive control) showed TUNEL positive signals in 75% of the nuclei. The strong FITC fluorescence was observed in the positive control, while TUNEL specific fluorescence was not observed in the negative control cells (a reaction mixture without terminal transferase was used). The frequency of TUNEL positive nuclei increased significantly compared to the control only in the roots that had been treated with 40 μM Al and reached 7.6% for 1-day treatment and 10.4% for 7-day treatment (Fig 10). Green fluorescence was observed in both the nuclei that were characterized by a normal morphology (Fig 9E, 9E`, 9F and 9F`) as well as in the fragmented nuclei (Fig 9G and 9G`) and micronucleated cells (Fig 9H and 9H`).

### The effect of Al on cell cycle

**Fig 11:**
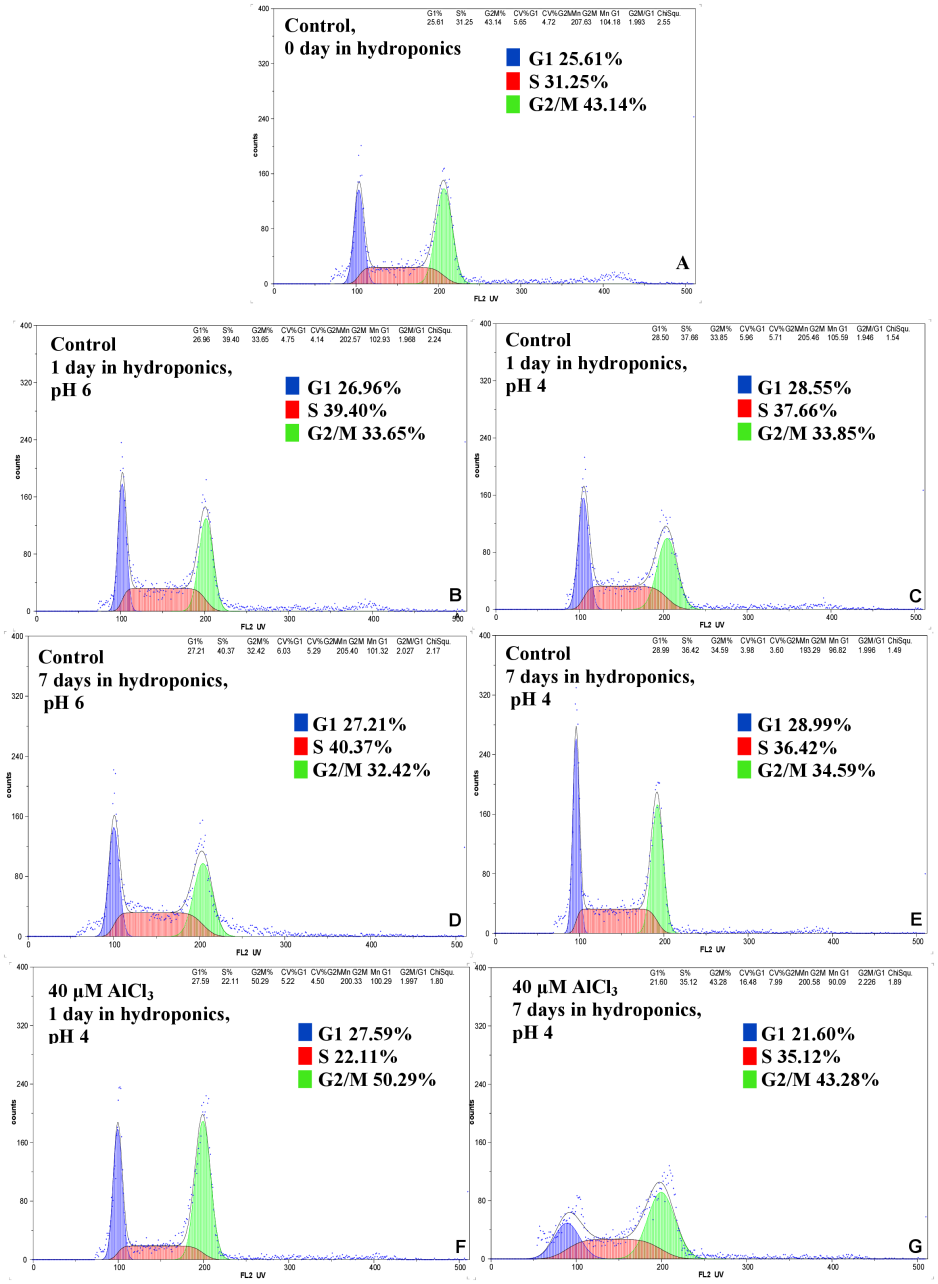
Flow cytometric analysis of cell cycle in roots of barley cv. ‘Sebastian’. (A–D) control roots, (E, F) Al treated roots.

To examine the effect of Al on the cell cycle in the barley meristematic root cells, an analysis using flow cytometry was performed (Fig 11). The cell cycle profiles of the control roots did not differ in the hydroponics on the 1^st^ and 7^th^ days of cultivation. Moreover, the pH of the hydroponic medium (pH 4 or pH 6) did not affect the frequency of cells in particular cell cycle phases. The frequencies of control root meristematic cells in the G1 phase were 26.96–28.99%, in the S phase 36.42–40.37% and in the G2 32.42–34.59% (Fig 11A–11D). These results indicate that the untreated barley root tip cells were predominantly in the S phase. After treatment with 40 μM Al, the root cells were predominantly in the G2/M phase (Fig 11F and 11G). The duration of treatment affected the frequency of G2/M cells. After 1-day treatment, the frequency of cells in S phase decreased drastically and 50.29% of cells were in G2/M, while after 7-day treatment only 43.28% of cells in G2/M were observed.

### The effect of Al on DNA replication

**Fig 12:**
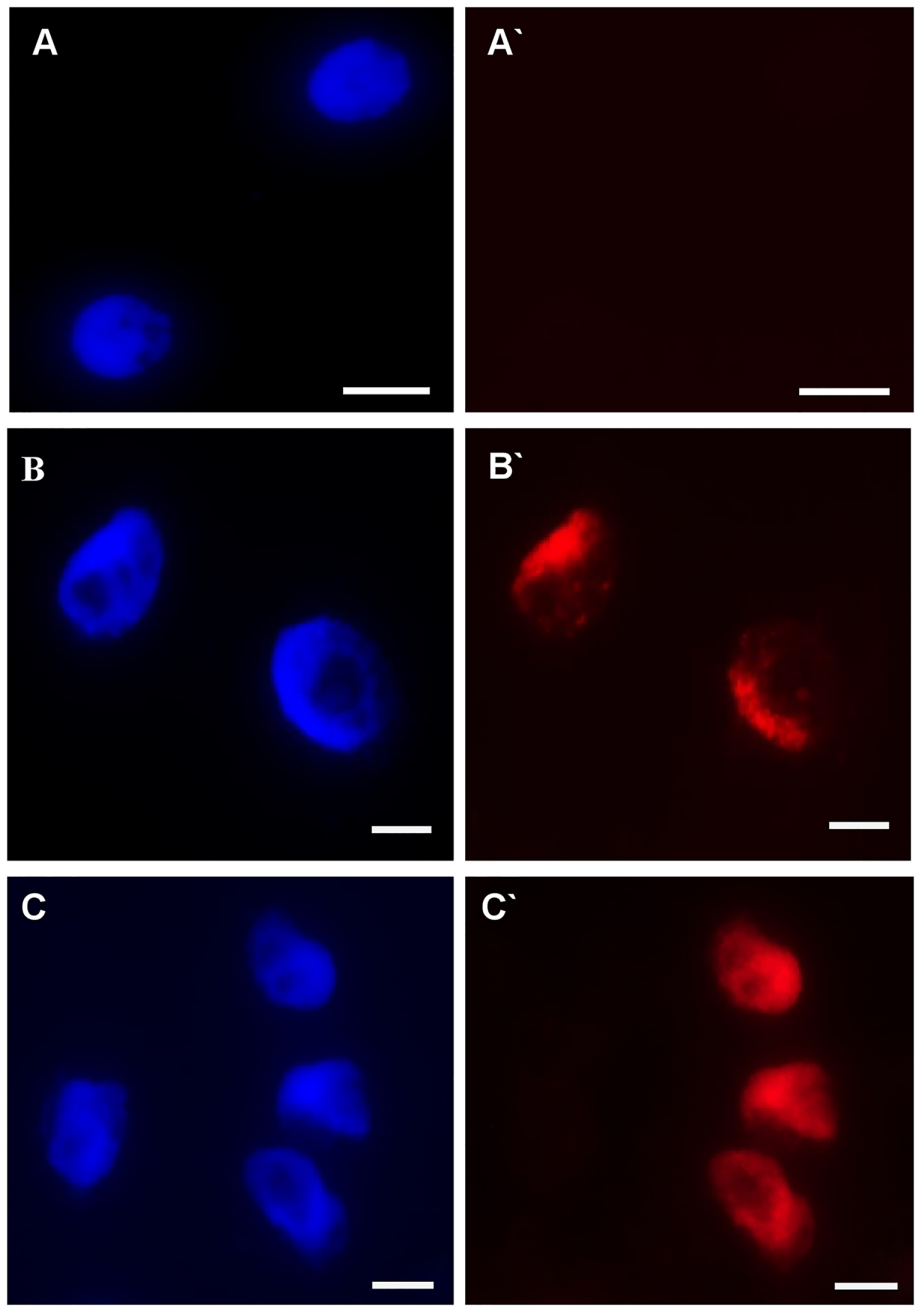
Examples of the detection of S-phase cells using EdU incorporation in roots of barley cv. ‘Sebastian’ seedlings treated with different concentrations of Al for 1 and 7 days. (A-C) DAPI staining (A`-C`) Alexa Fluor 647 fluorescence (red) in S-phase cells detected after EdU incorporation. Bars represent 20 μm.

**Fig 13:**
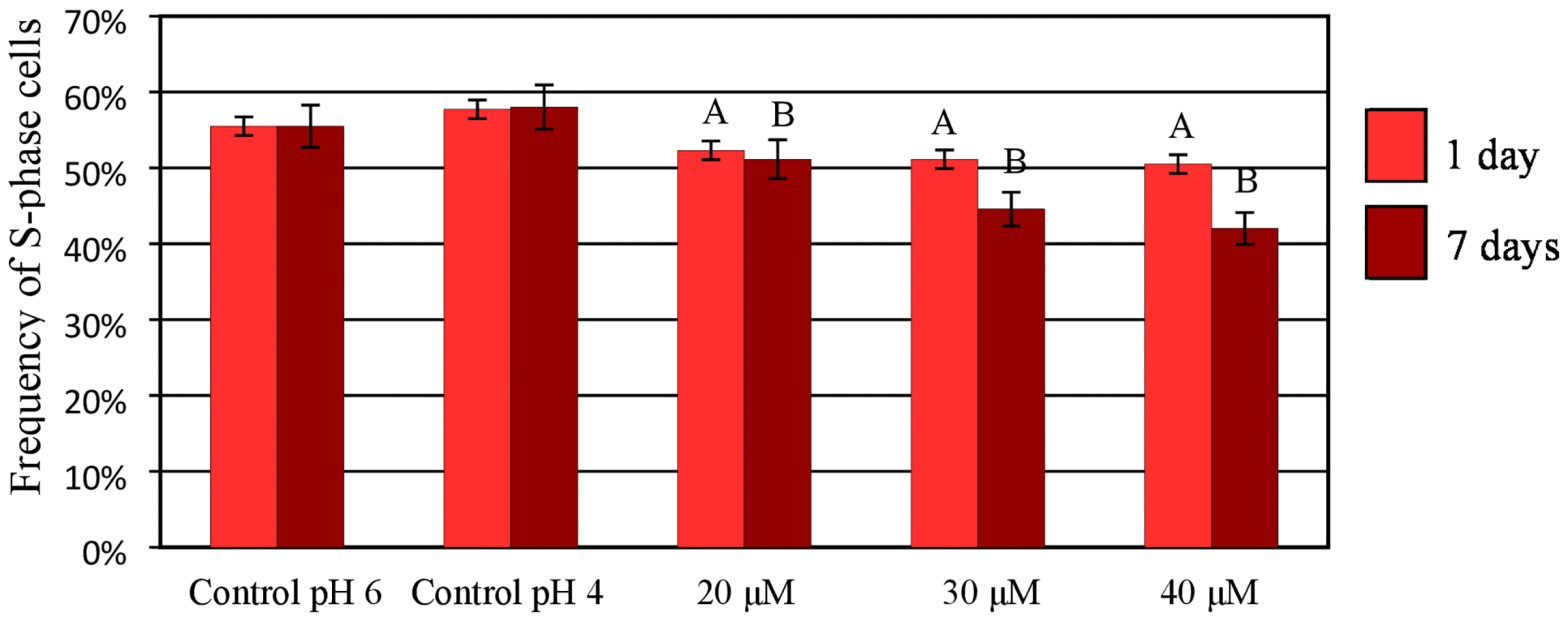
The frequency of root meristematic cells of barley cv. ‘Sebastian’ at S-phase in control and Al-treated roots. The error bars represent the standard deviations of the mean. The significant differences (P < 0.05) between the treated combinations and the controls (pH 4 and pH 6) within the same treatment day are indicated by a capital letter A (1-day treatment) or B (7-day treatment). The frequency of S-phase cells is not significantly different (P < 0.05) within the same treatment day for different pH.

To analyze the effect of Al on DNA replication in the nuclei of the barley root cells, the S-phase nuclei were identified using pulse labeling and of 5-ethynyl-2‘-deoxyuridine (EdU) was also detected. S-phase nuclei are visible as red marks in the Alexa Fluor 647 fluorescence; non-synthesizing DNA nuclei are presented as dim red (Fig 12). The possible effect of a low pH on the cell cycle was also analyzed for the control roots using a hydroponic media adjusted to pH 4 and 6. The frequencies of cells in particular cell cycle phases were similar, thus indicating no effects of pH on the frequency of cells in the S-phase (Fig 13). The treatment with 20, 30 and 40 μM Al significantly decreased the frequency of S-phase cells and thus confirmed the results of the flow cytometry analysis. The lowest frequency of S-phase cells (42%) was observed after treatment with 40 μM Al for 7 days.

## Discussion

The inhibition of root growth is the most obvious symptom of Al toxicity [32]. It is well known that this effect is linked to the cytogenetic symptoms that are related to the cell divisions upon exposure to Al [33]. We showed a significant decrease of mitotic activity in barley as a result of exposure to 20 μM Al. Previous studies showed a decrease in mitotic activity in other species, e.g. *Vicia faba* [34], *Allium cepa* [35] and *Helianthus annus* [36]; however, this was in response to higher doses of Al– 50 and 100 μM. There are several studies showing the different sensitivity of numerous barley varieties and cultivars to aluminum treatment [19]. However these results demonstrate the higher susceptibility of *Hordeum vulgare* cultivar ‘Sebastian’ to Al compared to other species. The mechanism that is responsible for the decreased cell division rate in roots after Al treatment may be connected to the direct Al binding to the DNA phosphate backbone [37,38]. The cytogenetic effects of Al treatment in barley that was observed in the presented study were compatible with the changes in the root growth parameters, such as decrease in the total root length, total root area and total root volume. Detailed analyses of the symptoms of root growth inhibition after Al treatment, which have not previously been reported for barley, were possible using a specialized root scanner coupled with the WinRHIZO software. These analyses are easy to handle and quick and therefore provide valuable data to predict the cytogenetic effects that are responsible for root inhibition, thereby replacing the time-consuming analyses in Al-optimizing experiments.

The impact of Al on DNA has already been suggested [38]. The effects of Al^3+^ ions on DNA integrity, which are observed as micronuclei, have been demonstrated in many species. Min et al. [39] reported a significant increase in the frequency of micronuclei in *Vicia faba* root tip cells after Al treatment in the range 0.01–10 mM. Chromosome aberrations induced by Al have also been reported in wheat [40] and rice [41]. Our results demonstrate that Al is a weak clastogenic agent in *Hordeum vulgare* cultivar ‘Sebastian’ cells that are exposed to the tested Al concentrations in a range of 20–40 μM. We also found that Al disturbed the morphology of nuclei, which has not previously been reported. This effect may be one of the symptoms of cell death that is induced by Al. The studies of Pan et al. [13] described some aspects of programmed cell death (PCD) and suggested that Al can lead to this process in barley and other plant species. Al-induced cell death has been studied in six cereal species including maize, wheat, triticale, rye, barley and oat [42]. DNA fragmentation, which was analyzed electrophoretically and indicated PCD, was observed in rye, barley and oat roots, but not in maize and wheat. These results suggest that wheat and maize are more tolerant to Al than the other analyzed species [42]. Data from our study using the TUNEL test confirmed that Al treatment induced DNA fragmentation in the barley root tip cells and therefore support this theory about PCD. The frequency of positively labeled nuclei in the TUNEL test was significantly different from the control only after treatment with 40 μM Al. As TUNEL-positive cells occurred more frequently than the disrupted nuclei, this fact may suggest that the DNA fragmentation that is induced by Al can be repaired and that not all TUNEL-positive nuclei become disrupted. Previous studies that used the comet assay showed that Al treatment resulted in an increase in DNA fragmentation thus indicating that Al directly affects DNA integrity in Arabidopsis roots [11]. No similar studies regarding the impact of Al on DNA integrity is known for barley.

Al has also been reported to delay cell divisions in root tips and inhibit DNA replication [32]. Recently, there has been a renewed interest in Al-induced alterations of the cell cycle, but most of these works are still focused on mitosis. Using flow cytometry analysis, we showed that after exposure to Al, the cell cycle profiles of the root tip cells differ from the profile of control roots. The frequency of cells in the G2/M phase increased after Al treatment and simultaneously the frequency of the S-phase cells decreased. Similarly, Doncheva et al. [32] reported a decrease of S-phase cells in maize after short-term Al exposure and therefore an inhibition of root cell divisions. The decreasing of the frequency of S-phase in barley was similar as in Al-resistant maize variety, whereas S-phase was completely stopped in Al-sensitive variety. Although it is evident that Al causes cell cycle disturbances, many aspects are still unknown, e.g. the species-specific dependence and reversibility of these changes remain to be elucidated in future experiments.

Detection of DNA synthesis in proliferating cells is possible through the incorporation of labeled DNA precursors into DNA during the S phase of the cell cycle. Nowadays, the click reaction with 5-ethynyl-2’-deoxyuridine (EdU) is applied in studies related to DNA damage and cell cycle disturbances [43]. In this study, the visualization of nuclei with DNA synthesis using EdU permitted the analysis of the effect Al on the DNA replication in barley root tips. The results confirmed the effect of Al treatment on the frequency of S-phase cells. It can be assumed that the cells did not enter the S phase as a response to Al. At the same time, the S-phase cells entered the G2/M phase, and therefore an increase in the frequency of cells in these phases was observed. The effects of Al have been studied in detail in Arabidopsis roots [23, 44–45]. To understand the Al impact on DNA damage and the cell cycle, a mutagenesis approach was used and resulted in the identification of Arabidopsis mutants with a hypersensitivity to Al. Using Arabidopsis mutants, it has been shown that Al causes the terminal differentiation of root tips and endoreduplication, together with a halting of the cell cycle progression in conjunction with a loss of the root-quiescent center [11,12].

The results of this study may help to understand the mechanism of Al action in barley cells. It is important to get know the processes that underlie Al toxicity under specific conditions including a species or cultivar sensitivity, medium composition, Al concentration and the duration of Al exposure.
